# Zoonotic potential of *Chlamydia psittaci*—a case report

**DOI:** 10.3389/fvets.2025.1638717

**Published:** 2026-01-20

**Authors:** Danijela Horvatek Tomić, Marija Krkljuš, Željko Gottstein, Liča Lozica, Estella Prukner-Radovčić

**Affiliations:** 1 Department of Poultry Diseases with Clinic, Faculty of Veterinary Medicine, University of Zagreb, Zagreb, Croatia; 2 Faculty of Veterinary Medicine, University of Zagreb, Zagreb, Croatia

**Keywords:** avian chlamydiosis, *C. psittaci*, zoonosis, parrot, child, respiratory infection

## Abstract

The causative agent of chlamydiosis/psittacosis, the obligatory intracellular bacterium *C. psittaci*, infects various species of birds and humans. Infected birds occasionally excrete the pathogen through the respiratory and digestive systems, with nasal/ocular discharge and feces being the main sources of infection for other birds or humans. Humans are most often infected through close contact with positive parrots. In this case report of avian chlamydiosis/psittacosis, samples were taken from a dead cockatiel and two budgerigars, and from a child living in the same household as the birds. In all the samples examined, except the child’s serum, *C. psittaci* was detected by real-time PCR. The phylogenetic analysis of the *ompA* gene from parrot isolates identified genotype A, confirming that parrots harbored the most virulent genotype of *C. psittaci*. To prevent the spread of avian chlamydiosis/psittacosis, it is necessary to procure birds from verified sources, monitor the signs of disease in parrots and humans, and strictly adhere to biosecurity measures to prevent further spread of the disease.

## 1 Introduction

Bacteria of the genus *Chlamydia* are the causative agents of significant diseases that occur in various species of animals and humans, and some also have zoonotic potential (1). These bacteria are characterized by their biphasic development cycle, producing elementary and a reticular body.

Avian chlamydiosis is an acute contagious infectious disease that belongs to neglected infectious diseases due to its complex etiology, transmission, and the ability to cause disease in different hosts (2). Natural hosts are birds, i.e., poultry, wild and pet birds. In addition to birds, the species *Chlamydia* (*C*.) *psittaci*, which is the most common cause of avian chlamydiosis, occurs in other animals such as cattle, sheep, goats, horses, pigs, dogs and foxes, and in humans, which makes this disease an anthropozoonosis (2). Several serovars and genotypes of *C. psittaci* were described (3). Comparing serotyping and genotyping, it was proven that the serovars correspond to the genotypes. The genotypes known so far are relatively highly specific. Genotype A is mostly associated with parrots, and most often causes disease in humans (2). Avian chlamydiosis can be asymptomatic, acute, subacute and chronic, and manifests as respiratory and gastrointestinal disease and/or as a systemic infection. The severity of the clinical signs depends on the age and species of the bird and the genotype causing the infection. Other factors that influence the occurrence of the disease include poor nutrition, overcrowding and stress (2). Acute infection usually affects younger birds, while in adults the disease is usually asymptomatic. In the acute stage of infection, symptoms of the upper respiratory system occur, such as discharge from the eyes and nose, lethargy, anorexia, hypothermia, and ruffled feathers. Diarrhea is present, usually yellow-green in color, and pericarditis, pneumonia, hepatitis, splenitis, and air saculitis may also occur (2). Pathoanatomical findings include hepatomegaly, splenomegaly, and fibrinous air sac inflammation (2). The prerequisite for successful diagnostics in birds is the sampling of different organs and/or in one or more consecutive days, and fast transport of samples to the laboratory. Most often, swabs of the conjunctiva, pharynx and/or cloaca and pooled fecal samples are preferable for routine diagnostics in birds, and organs swabs and serum in humans (4). Molecular methods commonly used are conventional PCR and real-time PCR, DNA-microarray test and sequencing (2). In the treatment of avian chlamydiosis, tetracyclines and fluoroquinolones are commonly administered (2).

To describe zoonotic potential of *C. psittaci*, this report presents a case of avian chlamydiosis/psittacosis in a child after contact with infected parrots.

## 2 Materials and methods

### 2.1 Case history

A dead female cockatiel (*Nymphicus hollandicus*), 6 months old, of unknown origin, was presented for necropsy to the Clinic of the Department of Poultry Diseases of the Faculty of Veterinary Medicine, University of Zagreb. There were already two budgerigars (*Melopsittacus undulatus*), 2 years old, without any clinical symptoms, living in the same house.

A month later, a 6-year-old child, living in the same household as the abovementioned parrots, developed respiratory symptoms. Clinical status shows that the child was subfebrile, conscious, in good general body condition, with normal respiratory murmur on the lungs. As there was a history of avian chlamydiosis in the household, a therapy with azithromycin *per os* was carried out for 5 days.

### 2.2 DNA isolation and PCR

DNA was isolated from the liver of the cockatiel and pooled fecal sample of the budgerigars, as well as from the nasal swab and serum of the child, using the GenElute Mammalian Genomic DNA Miniprep kit (Sigma-Aldrich, St. Louis, United States), according to the manufacturer’s instructions. The isolated DNA was stored at −20 °C until testing. DNA isolated from the liver and from the fecal sample were mixed and analyzed as one sample. The family *Chlamydiaceae* was identified by real-time PCR using specific primers and probes for the amplification of the 23S rRNA gene (5) and *C. psittaci* by using specific primers for the amplification of the *incA* gene (6). Each sample was run in duplicate, with a positive (*C. psittaci*) and negative control (sterile ultrapure water), also in duplicate. Samples were analyzed by using Mx3005P (Agilent Stratagene, La Jolla, United States) with the TaqMan fragment identification system, under the following conditions: 95 °C for 10 min, amplification for 50 cycles with denaturation at 95 °C for 15 s, a combined step of annealing and extension, during which the fluorescence was measured at 60 °C for 60 s (7).

### 2.3 Sequencing

To determine the genotype, sequencing of partial *ompA* gene of *C. psittaci* was performed (8). Sample was amplified using a Thermal Cycler 2720 (Applied Biosystems, Foster City, United States) under the following conditions: 95 °C for 2 min, amplification for 35 cycles with denaturation at 94 °C for 30 s, annealing at 60 °C for 60 s, extension at 72 °C for 60 s and final extension at 72 °C for 5 min (8). The products were visualized on a 1% agarose gel stained with Midori Advance (Nippon Genetics Europe GmbH, Düren, Germany). The fragment size was approximately 1,200–1,400 base pairs. Isolation and purification of DNA fragments from the agarose gel were performed according to the manufacturer’s instructions, using the ReliaPrep^™^ DNA Clean-up Concentration System kit (Promega, Madison, United States). The concentration and purity of the DNA was determined using BioDrop (Biodrop Ltd., Cambridge, United Kingdom), after which the samples were sequenced by the Sanger method by using Applied Biosystems 3500 Series sequencer (Macrogen Inc., Amsterdam, The Netherlands). The obtained sequence was analyzed using the software Molecular Evolutionary Genetics Analysis (MEGA12) 12.0.11 (9). Original phylogenetic tree was constructed by using maximum likelihood method and Tamura model of nucleotide substitutions (10) and *Chlamydia gallinacea* was used as outgroup.

### 2.4 Bacteriological and microscopical analysis

After the necropsy, cockatiel liver was examined by aerobic bacteriological examination. Both nutrient agar (Difco Nutrient Agar, Dickinson and Co., United States) and selective agar (Brilliant Green agar-modified, Oxoid, Great Britain) were used for the detection of aerobic bacteria. Plates were incubated at 38 °C for 24 h. Presumptive colonies were identified based on their morphological characteristics and Gram stain.

Pooled budgerigars’ fecal sample was examined to reveal the presence of *Macrorhabdus ornithogaster*. Briefly, pooled fecal sample was homogenized, smeared onto glass slide and stained by Gram stain (BioGram Eco, Biognost, Croatia) (11). Dried slide was visualized under the light microscope with 40× lens (Steinberg, Germany).

## 3 Results

The necropsy of the cockatiel showed hepato- and splenomegaly, although the owners did not report any clinical signs prior to death. Budgerigars were apparently healthy, showing no obvious clinical symptoms. Real-time PCR confirmed the presence of bacteria of the family *Chlamydiaceae* and the species *C. psittaci* in tested pooled liver and fecal sample. Single data points derived from real-time PCR amplification plots (threshold cycles-Ct) were 15.53/15.16 for *Chlamydiaceae* and 18.80/19.28 for *C. psittaci*, respectively.

Child clinical examination suspected psittacosis was confirmed by real-time PCR, detecting child nasal swab as positive for bacteria of the genus *Chlamydiaceae* and the *C. psittaci*, while the serum sample was negative. For child nasal swab, Ct values were 39.04/39.04 for *Chlamydiaceae* and 38.49/38.44 for *C. psittaci*.

**Figure 1:**
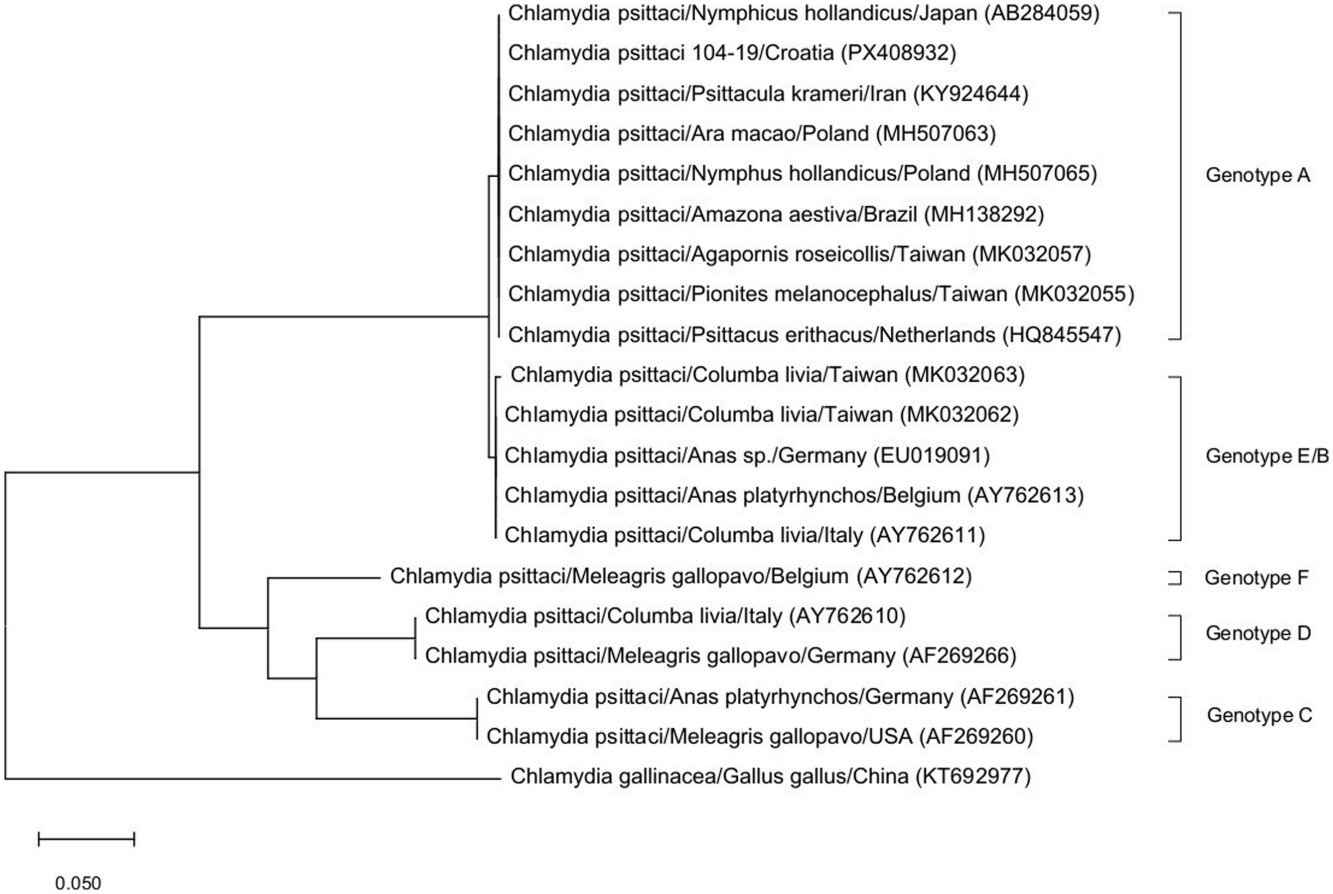
Phylogenetic analysis of the *ompA* gene sequence of *C. psittaci* originating from the parrots (104-19/Croatia; accession number PX408932) [maximum likelihood method and Tamura model (9)].

Analysis of the obtained *ompA* gene sequence and comparison with the reference sequences of different genotypes of *C. psittaci* available in the NCBI database encompassed 20 nucleotide sequences with 346 positions in the final dataset. Phylogenetic analysis revealed similarity (99.24%) to other isolates originating from parrots worldwide, with genotype A being confirmed (Figure 1). The sequence was deposited in the NCBI; accession number PX408932.

Although we were unable to perform the histopathological examination of the liver, it was tested for the presence of aerobic bacteria, and only *Staphylococcus* sp. and *Bacillus* sp. were isolated, while sample was negative for *E. coli* and *Salmonella*. Pooled fecal sample of the budgerigars was negative for *Macrorhabdus ornithogaster*.

The budgerigars were treated in accordance with regulations and under the supervision of the veterinary inspection with enrofloxacin for 21 days via drinking water, and the child was treated with azithromycin for 5 days orally. Repeated testing of the budgerigars yielded negative results for 2 weeks post treatment, and the negative finding was confirmed 10 months later, when the same budgerigars were re-tested for chlamydia. Data on the child’s recovery was not available.

## 4 Discussion

In this case report of avian chlamydiosis/psittacosis, liver and a pooled fecal sample were collected from a deceased cockatiel and two budgerigars, respectively, and tested positive for *C. psittaci*. Later, serum sample and nasal swab from a child living in the same household as the positive birds were taken due to the development of clinical symptoms, and child nasal swab also tested positive for *C. psittaci*.

As the liver of the dead cockatiel was examined together with a pooled fecal sample of the budgerigars, it was impossible to determine whether the main source of the chlamydia for the child was the newly acquired cockatiel or budgerigars, being already present in the household. Probably the source of *C. psittaci* was the newly purchased cockatiel which showed hepato- and splenomegaly, and not the budgerigars who tested negative 14 days post treatment and again 10 months later. As the only positive child sample was the nasal swab, it could be speculated that the child did not have a systemic infection. On the other hand, the fact that the child was subfebrile, in addition to the presence of the organism in the swab, indicated the presence of active (respiratory) infection. The possibility that positive nasal swab can be a consequence of the contaminated household or still positive budgerigars, after the death of the cockatiel, cannot be excluded, but as the budgerigars later tested negative twice, that was probably not the case. More reasonably, the source of *C. psittaci* was the newly purchased cockatiel which showed hepato- and splenomegaly, and not the budgerigars that have already been cohabiting with the child for 2 years previously, without detrimental health consequences for any of the household members.

In the case of avian chlamydiosis, transmission of the infection from bird to human occurs when humans are exposed to the pathogen, i.e., come into direct or indirect contact with infected birds (12, 13). It is important to emphasize that this disease is often asymptomatic in birds, as it was in this case, as they do not show clinical signs of the disease, but are still a possible source of infection.

Molecular methods are recommended for the diagnosis of chlamydia because they are rapid, sensitive and specific (2). Such methods also enable detection of probable sources of infection and assist in the reporting, surveillance and management of the outbreaks (14). Based on the sequencing of the *ompA* gene, *C. psittaci* isolate from parrots were identified as genotype A (Figure 1). This finding coincides with those presented by Heddema et al. (15), who proved that genotypes A and B of *C. psittaci* were the most common causes of psittacosis in the Netherlands. A similar study was conducted by Gedye et al. (16) who also found that two genotypes (A and C) of *C. psittaci* were most commonly present in domestic and imported birds in New Zealand, and that both genotypes are globally associated with significant disease in birds and humans. Unfortunately, comparison of bird- and human-derived *C. psittaci* genotypes could not be done in this case, because complete sequencing of the child’s isolate was not feasible. However, the fact that *C. psittaci* was detected in birds as well as in a child from the same household, and that one bird died while the child was subfebrile and required therapy a month later, makes the presentation of this case relevant. It is also worth mentioning that it is often difficult to determine the origin of the infection or the family context, with identifiable exposure to birds. Also, the small amount of chlamydial DNA in human samples, especially when the samples are taken non-invasively (as was the child nasal swab in this case), which is used for chlamydia detection, makes sequencing and genotyping difficult (17).

As psittacosis is not significantly different from other respiratory diseases, patients should mention that they have been in contact with birds. Due to low awareness of the disease itself and the variable clinical signs, physicians often fail to recognize psittacosis. Therefore, it is necessary to mention the possibility of infection with *C. psittaci* if there is a pet bird in the same household or professionally being in close contact with birds. From veterinary point of view, the strict quarantine for all newly acquired birds is essential, in order to prevent the transmission of infectious diseases.

## 5 Conclusion

Avian chlamydiosis is not common zoonotic disease, but can be detected in different bird species, such as parrots, especially if the birds are not properly kept, or are under stress. Humans that are often in contact with different bird species, like bird owners, veterinarians etc., are most commonly infected with *C. psittaci*. To prevent the spread of infection to other birds or humans, it is necessary to purchase birds from trusted sources, adhere to biosecurity measures, maintain cage hygiene, and regularly remove droppings. If the disease does occur in birds, it is necessary to comply with all prescribed legal measures, report suspicion or confirmation of the disease itself, and carry out treatment and disinfection. In the event of psittacosis, it is necessary to mention contact with any type of bird to the medical professional.

## Data Availability

The authors acknowledge that the data presented in this study are deposited and publicly available in an acceptable repository, prior to publication. The sequence was deposited in the NCBI; accession number PX408932.
